# Perceived Value in Specialty Coffee: A Means-End Chain Model Applied in the Brazilian Context

**DOI:** 10.3390/foods15122220

**Published:** 2026-06-19

**Authors:** Ari Melo Mariano, Gustavo Garcia Loguercio, Ingrid Orlandini, Carla Patricia Pareja-Daza, Maíra Rocha Santos

**Affiliations:** 1 DataLab-Department of Production Engineering, Universidade de Brasília, Brasilia 70910-900, Brazil; 2 Department of Production Engineering, Universidade de Brasília, Brasilia 70910-900, Brazil; 3 Faculty of Economic and Business Sciences, Universitaria San Francisco Xavier de Chuquisaca, Sucre 212, Bolivia; 4 Department of Design, Institute of Arts, Universidade de Brasília, Brasilia 70910-900, Brazil

**Keywords:** specialty coffee, perceived value, PLS-SEM, Brazil

## Abstract

This study aims to identify, based on a Means-End Chain model, the main linkages among attributes, benefits, and perceived value in specialty coffee. Specialty coffee, recognized for its superior quality and sustainable production practices, has experienced growing demand in Brazil and worldwide, intensifying competition and increasing the need to understand how value is constructed from the consumer’s perspective. A quantitative study was conducted using PLS-SEM to test a model adapted to the specialty coffee context, applied to a sample of 88 respondents. The model was validated and explained 29.4% of the variance in Perceived Value, highlighting the role of Attributes and particularly of Benefits and Consequences as the cognitive link that transforms product characteristics into perceived value. In addition to direct effects, significant indirect effects were identified, indicating that a substantial portion of the impact of attributes occurs through mediation, influencing particular, convenient, and rational benefits before being reflected in perceived value. The findings contribute to a systemic understanding of how elements of the coffee value chain are cognitively connected in consumers’ minds and provide insights for positioning, communication, and differentiation strategies to promote sustainable, high-quality coffees.

## 1. Introduction

Brazil is the world’s largest producer and exporter of coffee, accounting for approximately 30% of global production, according to data from the International Coffee Organization [1]. In 2022, the country produced approximately 171.3 million 60 kg bags of coffee, with an estimated gross value of R$ 50 billion [2]. The coffee sector is essential to the Brazilian economy, employing millions of people and contributing significantly to the country’s agricultural GDP. Historically, the coffee market has evolved in consumption waves that reflect changes in consumer preferences, ranging from mass production in the 1960s to the popularization of specialty coffees beginning in the 1990s, and more recently to the appreciation of small producers and sustainable practices in the so-called third wave, which emerged in the 2000s [3].

Specialty coffees, as defined by the Specialty Coffee Association, are those that achieve a minimum score of 80 points on a 100-point scale. They stand out for superior quality and production practices that emphasize sustainability and social responsibility [4]. This segment has experienced remarkable growth, driven by consumers who seek not only sensory quality but who are also increasingly aware of the environmental and social impacts of their consumption choices [5]. Likewise, the global specialty coffee market is projected to continue expanding, with forecasts indicating annual growth of approximately 11.3% through 2030 [6].

As highlighted in previous studies [7,8,9], the consumer-perceived value of specialty coffee is influenced by a combination of factors. These include sensory attributes, sustainability, innovations within the production chain, quality management, and communication strategies. In the current scenario, competition has intensified with the diversification of high-quality coffee offerings, requiring producers to invest in differentiation strategies in order to capture consumer interest [8].

Research on specialty coffee consumption has advanced along three main streams: sensory and experimental studies, which show how roasting profiles, cup texture, and packaging information shape hedonic judgments and willingness to pay [8,10,11]; behavioral and acceptance models that explain purchase intention and adoption across countries [9]; and work on sustainability along the coffee value chain, covering circular-economy barriers and enablers [12], sustainability visions for the industry [5], and the consumption practices of connoisseur consumers [13]. This progress has not closed three gaps. Attributes, benefits, and values are usually investigated in isolation, so the complete cognitive chain that links them is rarely modeled. Quantitative tests of structural models based on the Means-End Chain (MEC) theory also remain scarce in the coffee domain; the main antecedent, the capsule-coffee model of Oliveira et al. [14], was not developed for the specialty segment. Evidence from producing countries such as Brazil, finally, is still limited when compared with studies conducted in consuming markets.

This study addresses these gaps by adapting the MEC-based model of Oliveira et al. [14] to specialty coffee and testing it empirically with Brazilian consumers using partial least squares structural equation modeling (PLS-SEM). Its main contributions are (i) an integrated, simultaneous estimation of the attribute–benefit–value chain rather than the analysis of isolated relationships; (ii) the explicit incorporation of sustainability, innovation, and quality dimensions into the benefit structure of the model; and (iii) evidence on the mediating role of benefits and consequences, clarifying the mechanism through which product attributes are transformed into perceived value.

From a production engineering perspective, this research is justified by the need to optimize production processes and market strategies within the specialty coffee landscape. Efficient methods can thus be developed to add value to the product, enhance competitiveness, and support strategic decision-making that aligns production, logistics, and quality management.

Therefore, the guiding research question is as follows: How are the different specialty coffee factors and their influences on consumer-perceived value related? The objective of this study is to identify, based on a Means-End Chain model, the main linkages among attributes, benefits, and perceived value in specialty coffee. In simple terms, the study investigates which characteristics of specialty coffee matter to consumers, how these characteristics are translated into perceived benefits, and how such benefits build the value that consumers attribute to the product.

The remainder of this paper is structured as follows: First, the theoretical foundation is presented. Next, the study methodology is described. Subsequently, the results are analyzed and presented, followed by the discussion.

## 2. Background

Coffee is one of the most widely consumed beverages in the world. According to the International Coffee Organization’s Coffee Market Report for October 2023, 168.5 million 60 kg bags were produced during the 2021/2022 crop year, and production is expected to reach 171.3 million bags in the 2022/2023 coffee year, representing an increase of 1.7% [1]. In addition, global coffee consumption has increased over the years and remains in a recovery phase following the COVID-19 pandemic. Accordingly, consumption of more than 178.5 million bags is projected for 2022/2023 [1].

Specialty coffee, or specialty-grade coffee, refers to a category that meets the quality criteria established by the Specialty Coffee Association. Following SCA methodologies, such as the Cupping Method, coffee is classified as “specialty” if it achieves a minimum score of 80 out of 100 points. In this sense, specialty coffee is also understood as a premium or gourmet product that embodies the philosophy of enhancing consumer satisfaction through superior bean quality and refined preparation techniques [9].

In Brazil, specialty coffees have been gaining an increasing market share. According to research conducted by the Associação Brasileira da Indústria de Café in 2022, sales of high-quality coffee categories showed significant growth in volume, with a 26% increase in the superior category, 20% in the gourmet category, and 5.5% in capsule coffee [15].

### 2.1. Means-End Chain (MEC) Theory

**Figure 1:**
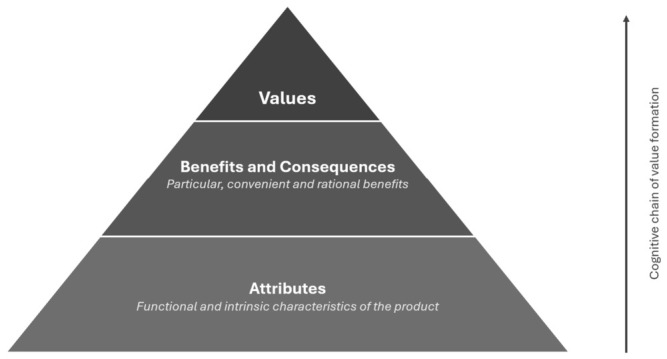
Means-End Chain (MEC) theory. Source: Authors’ own elaboration.

To operationalize the factors described, this study is grounded in the model proposed by Oliveira et al. [14], drawing on the Means-End Chain (MEC) theory and the assumption that choices are guided by Attributes, Consequences, and Values (A-C-V). Accordingly, it is feasible to operationalize the research using the Attributes–Consequences–Values (A–C–V) framework, as illustrated in Figure 1.

According to Oliveira et al. [14], particular benefits refer to advantages directly associated with the individual consumer’s experience, such as satisfaction and personal fulfillment derived from product use. Convenient benefits relate to the practicality and ease the product provides, enhancing everyday life and simplifying tasks associated with its use. In contrast, rational benefits involve a more objective, logical evaluation, linking product use to concrete outcomes such as efficiency, cost savings, or long-term impacts, thereby justifying the choice based on rational, strategic criteria. These three categories of benefits help explain how consumers connect product attributes to their purchase decisions and personal values.

Within the Means-End Chain (MEC) theory, it is argued that tangible product attributes generate consequences that, in turn, lead to values. Functional attributes are product characteristics that directly influence a product’s function or performance. Conversely, intrinsic attributes are the specific or inherent characteristics of a product, namely the particularities that differentiate it from others in the market.

### 2.2. Value in the Production Process

The production process of specialty coffee is essential for adding value to the product, with a focus on sensory quality and sustainability throughout the entire production chain, from cultivation to roasting and preparation. As discussed by Eiseman and Jonsson [16], advanced techniques such as controlled fermentation and environmental monitoring are employed to enhance sensory attributes, including aroma and flavor, which consumers highly value. In this context, efforts to improve raw material quality are achieved through rigorous quality control practices, including manual bean selection and careful post-harvest processing [8].

Environmental and social responsibility within the production process play a crucial role in value generation. The adoption of sustainable agricultural practices, such as agroforestry systems and reduced chemical inputs, improves coffee quality while responding to the growing demand for more responsible products [17]. Consequently, certifications from recognized institutions reinforce the added value by communicating a commitment to ethical and sustainable practices, thereby differentiating products in the global market [9].

Considering the contextualization and elements previously presented and based on the studies conducted and referenced, a structural model was developed comprising different hypotheses aimed at explaining consumers’ perceived value of specialty coffee. Functional and intrinsic attributes and their relationships with product consequences and benefits were evaluated. Three key factors showing the strongest alignment with specialty coffee benefits were selected for further analysis.

**Figure 2:**
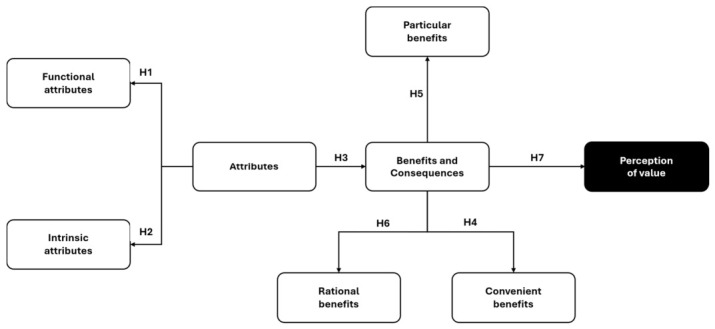
Structural model. Source: Adapted from Oliveira et al. [14].

Accordingly, the following structural model is proposed to correlate the different perspectives (Figure 2), with the first two research hypotheses formulated below.

The literature indicates that tangible and intrinsic product attributes constitute the foundation of consumers’ initial evaluations. Functional attributes refer to characteristics related to accessibility, ease of handling, and available variety, whereas intrinsic attributes concern inherent qualities such as flavor, aroma, and quality score.

According to Oliveira et al. [14], within the Attributes/Consequences/Values framework, attributes represent the first level of the cognitive chain leading to value formation. Furthermore, Barahona et al. [10] emphasize that sensory characteristics and product-related information directly influence consumer evaluation. Accordingly, the following hypotheses are proposed:

**H1.**  *Specialty Coffee Attributes positively influence Functional Attributes.*

**H2.**  *Specialty Coffee Attributes positively influence Intrinsic (Own) Attributes.*

The Means-End Chain theory posits that attributes do not generate value directly, but rather through perceived consequences [14]. In the context of specialty coffee, attributes related to quality, origin, and sensory differentiation activate perceptions associated with experience, sustainability, and identity.

Giacalone et al. [8] and Carvalho et al. [11] demonstrate that sensory and contextual elements are cognitively interpreted, while Van Keulen and Kirchherr [12] indicate that sustainable practices within the value chain influence consumer evaluation. Thus, the following hypothesis is proposed:

**H3.**  *Specialty Coffee Attributes positively influence the Benefits and Consequences perceived by consumers.*

Perceived Benefits and Consequences unfold into three main dimensions within the model: Particular Benefits, Convenient Benefits, and Rational Benefits.

Particular Benefits are associated with individual satisfaction, well-being, and emotional connection. Ramírez-Correa et al. [9] indicate that specialty coffee consumers seek differentiated experiences and authenticity, reinforcing the experiential dimension.

Convenient Benefits are related to innovation, practicality, and personalization, as discussed by Suryani et al. [7], who highlight the relevance of innovation to performance in the coffee sector.

Rational Benefits refer to sustainability, socio-environmental responsibility, and the ethical impact of consumption, aligning with discussions by Van Keulen and Kirchherr [12], Chemura et al. [17], and Beske and Seuring [18] regarding sustainable value chains.

Based on this evidence, the following hypotheses were formulated:

**H4.**  *Benefits and Consequences positively influence Convenient Benefits.*

**H5.**  *Benefits and Consequences positively influence Particular Benefits.*

**H6.**  *Benefits and Consequences positively influence Rational Benefits.*

The final stage of the model corresponds to the formation of Perceived Value. According to Oliveira et al. [14], value emerges from the interpretation of consequences associated with attributes. In the context of specialty coffee, this perception is influenced by sensory, experiential, and sustainability-related dimensions [10,13].

Accordingly, the following hypothesis is proposed:

**H7.**  *Perceived Benefits and Consequences positively influence Consumer-Perceived Value regarding specialty coffee.*

Thus, the proposed structural model integrates sensory quality, innovation, and sustainability within a hierarchical structure grounded in Means-End Chain theory. The model will be estimated and presented in the Section 4. Read without the technical vocabulary, the model says that consumers first form an overall evaluation of specialty coffee attributes, expressed in functional aspects, such as availability, access, and ease of use, and in intrinsic aspects, such as flavor, aroma, and quality score (H1 and H2); this evaluation activates the benefits and consequences that consumers perceive (H3), which unfold into particular, convenient, and rational benefits (H4–H6) and are converted, at the end of the chain, into the value attributed to specialty coffee (H7).

## 3. Materials and Methods

For the development of this research and the achievement of the proposed objectives, an explanatory research design was adopted, prioritizing the identification of factors that determine or contribute to the occurrence of the phenomena under investigation, with the aim of explaining the underlying reasons and causal relationships [19].

Regarding procedures and techniques, a quantitative approach was defined, employing structural equation modeling as the primary analytical method.

### Instrument and Measurement Scale

For the development of the data collection instrument (Table 1), items were adapted from Oliveira et al. [14]. The instrument consisted of 26 questions distributed across six variables: Intrinsic (Own) Attributes (AP), comprising 2 items; Functional Attributes (AF), with 3 items; Convenient Benefits (IN), with 3 items; Particular Benefits (QU), with 6 items; Rational Benefits (SU), with 4 items; and Perceived Value (V), with 8 items. Item codes follow the notation of the original instrument [14].

A five-point Likert scale was used as the measurement criterion. Response options ranged from “Strongly Agree,” “Partially Agree,” “Neither Agree nor Disagree,” “Partially Disagree,” to “Disagree.”

The sample size was calculated using G*Power 3.1.9.7 based on effect size estimation. A medium effect size (f^2^ = 0.15) was assumed, with a statistical power of 0.80 and a significance level (α) of 0.05, considering two predictors [20]. The calculation indicated a minimum required sample of 68 respondents. A total of 88 valid responses were ultimately obtained. The adequacy of this sample was also verified through the inverse square root method [21], according to which a sample of 88 respondents provides 80% statistical power, at the 5% significance level, to detect standardized path coefficients of 0.265 or higher (2.486/√88); as reported in Section 4, all hypothesized paths exceeded this threshold. Although modest in absolute terms, the sample size is therefore sufficient for the inferential purposes of the model, a property of PLS-SEM that has been demonstrated even under small-sample conditions [21,22,23].

The instrument was administered via Google Forms and disseminated via social media and professional contact networks. Sampling was therefore non-probabilistic, following a convenience strategy with snowball dissemination. Eligibility was restricted to adults (18 years or older) residing in Brazil; because dissemination occurred within networks linked to coffee interest and consumption, respondents are predominantly specialty coffee consumers, although consumption frequency was not used as a screening criterion. The questionnaire opened with an informed-consent statement, responses were collected anonymously, and all items were mandatory, which prevented missing data; accordingly, all 88 completed questionnaires were retained for analysis. Because participation was voluntary, the sample is subject to self-selection and is not statistically representative of the Brazilian population; its concentration of highly educated, higher-income respondents is characteristic of the specialty coffee niche but bounds the scope of the conclusions, as acknowledged in the limitations. To mitigate common method concerns at the design stage, the procedural remedies recommended by Podsakoff et al. [24] were adopted, including respondent anonymity, assurance that there were no right or wrong answers, and the organization of scale items into separate construct blocks.

The collected data were analyzed using partial least squares structural equation modeling (PLS-SEM) in SmartPLS 4.0, which simultaneously estimates the measurement model (relations between latent variables and their indicators) and the structural model (relations among latent variables), assessing whether the empirical data are consistent with the theoretical framework under investigation and identifying which constructs exert the strongest influence on perceived value. PLS-SEM was preferred over covariance-based SEM (CB-SEM) for four reasons [22,23,25]: (i) the purpose of the study is explanatory and predictive, namely estimating the strength of the attribute-benefit-value chain, rather than testing the covariance structure of a consolidated theory; (ii) the model is relatively complex for the available sample, and PLS-SEM achieves higher statistical power under small-sample conditions; (iii) PLS-SEM does not require multivariate normality, which is appropriate for Likert-type data collected online; and (iv) the model is an adaptation of an existing framework to a new empirical context, a typical application of composite-based estimation [22,23]. Statistical inference was based on bootstrapping with 5000 subsamples, using bias-corrected and accelerated (BCa) confidence intervals and two-tailed tests at the 5% significance level [22]. In addition to the procedural remedies described above, common method bias was assessed statistically through collinearity diagnostics: variance inflation factor (VIF) values below the conservative threshold of 3.3 indicate that the model can be considered free of substantial common method contamination [26]. Finally, because the survey link was disseminated openly, a classical non-response rate cannot be computed; the associated risk of self-selection bias is acknowledged and discussed in the limitations [27].

## 4. Results

The sample consisted of Brazilian adults who had reached the age of majority. The respondents’ profile included 65.9% men and 34.1% women, with an average age of 50 years. Regarding marital status, 60.2% of participants were married. Regarding education, 48.9% had completed higher education. The sample also exhibited a relatively high income level by Brazilian standards, with 42% reporting a monthly income above R$20,000.

Rather than constituting a finding in itself, this profile delimits the population to which the results can be extrapolated: the model was estimated on a niche of mature, highly educated, higher-income consumers, which is coherent with prior characterizations of premium and sustainable product audiences [28]. The implications of this concentration for the generalizability of the results are examined in the limitations section.

**Figure 3:**
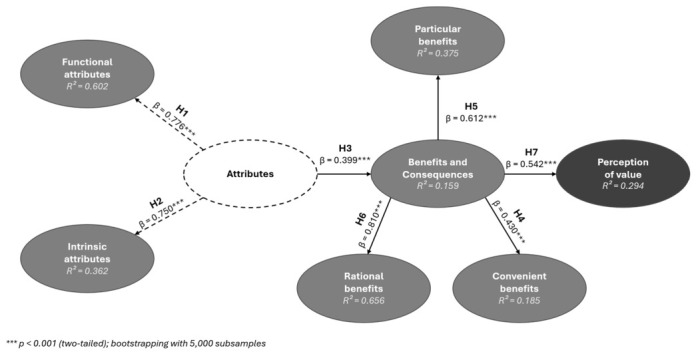
Estimated structural model: standardized path coefficients (β) and coefficients of determination (R^2^). Source: Authors’ own elaboration, extracted from SmartPLS 4.1.1.8 (2026).

For the application of the structural equation modeling (SEM) approach, the Means-End Chain (MEC) theory [14] was adopted as the theoretical foundation. Based on this framework, and drawing on the studies by Van Keulen and Kirchherr [12] and Giacalone et al. [8], eight variables were specified within a reflective measurement model, as illustrated in Figure 3.

### 4.1. Measurement Model

#### Reliability and Validity of the Model

Indicator reliability was examined using the outer loadings of the reflective measurement model. Outer loadings of 0.707 or greater indicate adequate indicator reliability [25]; however, values between 0.40 and 0.708 may be retained provided that their removal does not improve composite reliability or convergent validity, whereas indicators below 0.40 should always be eliminated [22]. These criteria guided item retention in the present study. In the initial estimation, indicators IN1, QU1, QU4, and QU6 presented insufficient indicator reliability (initial loadings between 0.256 and 0.699) and were removed during measurement purification; indicator V8 was likewise excluded during instrument refinement. The final measurement model therefore retained 21 of the 26 original items, with outer loadings ranging from 0.637 to 0.911; the few indicators below the 0.708 reference (V4 to V7) were retained because the composite reliability and convergent validity of Perceived Value remained adequate [22]. The complete matrix of initial and final outer loadings per construct is reported in Appendix A (Table A1) (Figure 3).

Additionally, composite reliability values (ρc) greater than 0.70 demonstrate satisfactory internal consistency of the constructs. On the other hand, Average Variance Extracted (AVE) values above 0.50 indicate adequate convergent validity, as they demonstrate that the construct explains more than half of the variance of its indicators [29] (Table 2).

To assess discriminant validity, the Heterotrait-Monotrait ratio (HTMT) was used as the criterion. To ensure adequate discriminant validity, HTMT values should be less than or equal to 0.90, as recommended by Henseler et al. [30] (Table 3).

The final stage in assessing the measurement model involves analyzing multicollinearity by calculating the Variance Inflation Factor (VIF). This procedure aims to determine the extent to which the standard error is inflated by collinearity among indicators.

According to Hair et al. [31], VIF values should not exceed 3.3; otherwise, indicator removal may be necessary. Additionally, Ramírez et al. [25] indicate that VIF values greater than 10 clearly signal a serious multicollinearity problem. In the present model, all VIF values remained below the conservative threshold of 3.3, indicating the absence of substantial collinearity among indicators. Following the full collinearity rationale of Kock [26], VIF values at or below 3.3 also support the conclusion that the model is not substantially contaminated by common method bias, complementing the procedural remedies described in Section 3.

### 4.2. Structural Model

Beta values considered ideal are β ≥ 0.30 or β ≤ −0.30. However, values of β ≥ 0.20 or β ≤ −0.20 may also be considered meaningful in the context of the analysis [32]. In conjunction with the evaluation of beta coefficients, bootstrapping analysis was performed to ensure the stability of sample estimates. This procedure allows assessment of statistical significance using the t-value (Student’s t) and the *p*-value. The thresholds commonly adopted to ensure confidence in the model are t ≥ 1.64 and *p* < 0.05. As described in Section 3, the bootstrapping procedure was executed with 5000 subsamples, bias-corrected and accelerated (BCa) confidence intervals, and two-tailed tests at the 5% significance level; the resulting 2.5% and 97.5% confidence bounds are reported alongside each path estimate. The values obtained in this study are presented in Table 4.

It can be observed that all hypotheses were supported, as they presented t-values greater than 1.64 and *p*-values lower than 0.05, indicating statistically significant relationships within the proposed structural model.

Hypothesis H1 proposes that Attributes positively influence perceived Functional Attributes. This effect is particularly evident in the specialty coffee market, where functional aspects such as preparation speed and consistency of quality are valued by consumers seeking convenience without sacrificing excellence [12]. Similarly, Mazwan et al. [33] emphasize that consumers value products that meet their functional needs while aligning with their personal values. Therefore, to maximize perceptions of functional attributes, firms increasingly adopt integrated approaches that combine efficiency, quality, and responsible practices, thereby creating a stronger, more differentiated appeal in the specialty coffee market.

Hypothesis H2 suggests that perceived general attributes positively influence the intrinsic attributes of specialty coffee, such as flavor, aroma, and sensory complexity. A high-quality perception of general attributes enhances the appreciation of intrinsic characteristics, which are essential for the distinctive sensory experience sought by specialty coffee consumers [10]. In a competitive market, differentiation is fundamental for customer loyalty. In this context, informed and demanding consumers tend to value coffees with unique sensory profiles, fostering emotional connections and increasing their willingness to pay for authentic, differentiated experiences [9].

Regarding Benefits and Consequences (H3), practices such as consumption personalization allow consumers to select specific beans based on their sensory preferences and personal values. This expanded perception of attributes may also stimulate the development of complementary products, such as biodegradable capsules or brewing accessories that enhance sensory characteristics. Hypothesis H4 posits that consumers value convenient benefits, including transparency, experimentation, diversity, variety, creativity, and personalization [12]. Likewise, Carvalho et al. [11] show that product innovation, such as infused coffees or differentiated roasting techniques, adds value. Personalization is further enhanced by technologies that adjust brewing parameters according to individual preferences.

Hypothesis H5 proposes that perceived Benefits and Consequences positively influence Particular Benefits, such as individual satisfaction, well-being, and emotional connection with the product. These benefits are crucial for specialty coffee consumers who seek not merely a beverage, but an enriching experience aligned with their personal values [17]. Particular benefits are perceived when coffee meets high-quality standards and reflects values such as sustainability and authenticity. This suggests that consumers show strong interest in high-quality products, product differentiation, and closer connections with producers.

Hypothesis H6 reflects the growing consumer interest in products that combine quality with social and environmental responsibility. Consumers who recognize the positive impact of their choices, such as sustainability within specialty coffee value chains, tend to value these Rational Benefits and incorporate them into their purchasing decisions [8]. In this market, rational benefits, including perceived sustainability and economic efficiency, are valued because they extend beyond the product itself and address ethical and practical considerations that resonate with consumer values [13].

Finally, Hypothesis H7 suggests that perceived Benefits and Consequences positively influence consumers’ Perceived Value. A comprehensive and positive evaluation of overall benefits, including convenience, sustainability, and personal experience, results in higher value perception, strengthening the consumer-product connection and decisively influencing purchase decisions [10]. Similarly, Oliveira et al. [14] converge on this conclusion, confirming the positive influence of Benefits and Consequences on consumers’ Perceived Value.

From an explanatory standpoint, the model accounts for distinct shares of variance across the endogenous constructs (Figure 3): 60.2% of Functional Attributes, 56.2% of Intrinsic (Own) Attributes, 65.6% of Rational Benefits, 37.5% of Particular Benefits, 18.5% of Convenient Benefits, 15.9% of Benefits and Consequences, and 29.4% of Perceived Value. All R^2^ values exceed the 10% minimum recommended for soft modeling [29]. According to the classification of Chin [32], the explanatory power obtained ranges from weak (Convenient Benefits and Benefits and Consequences) to close-to-substantial (Rational Benefits and Functional Attributes), with Perceived Value falling between the weak and moderate bands. These results reinforce the hierarchical structure proposed by the Means-End Chain theory, but they also indicate that part of the variability of convenience- and value-related perceptions is driven by factors outside the model; this point is examined critically in the Discussion.

### 4.3. Indirect Effects and the Mediating Role of Benefits and Consequences

The analysis of indirect effects revealed that Attributes do not impact subsequent constructs solely through direct relationships; they also exert a significant influence, mediated by the Benefits and Consequences variable.

The results indicate that all indirect paths were statistically significant, demonstrating that Benefits and Consequences function as a central mediating variable within the structural model. Regarding the type of mediation, the structural model, following the hierarchical logic of the Means-End Chain theory, does not specify direct paths from Attributes to the three benefit dimensions or to Perceived Value; consequently, the significant indirect effects reported in Table 5 are characterized as indirect-only mediation, the pattern consistent with full mediation in the typology of Zhao, Lynch, and Chen [34]. In other words, the entire effect of Attributes on Particular, Convenient, and Rational Benefits and on Perceived Value is transmitted through Benefits and Consequences. Concerning magnitude, the standardized indirect effects ranged from 0.171 (Convenient Benefits) to 0.323 (Rational Benefits), values conventionally interpreted as small-to-moderate effects [20,32]; the strongest indirect effect converges on the rational, sustainability-related dimension, mirroring the pattern observed for the direct paths.

## 5. Discussion

The results obtained allow for Perceived Value in the specialty coffee market to be understood as a systemic phenomenon, structured by a cognitive chain that articulates attributes, benefits, and values, in accordance with the Means-End Chain theory. The model’s statistical robustness, as evidenced by reliability, discriminant validity, absence of multicollinearity, and satisfactory coefficients of determination, reinforces the empirical consistency of the proposed structure.

The model explains 29.4% of the variance in Perceived Value, a percentage considered relevant in complex behavioral models. Particularly noteworthy are the high explanatory levels of Rational Benefits (R^2^ = 65.6%) and Functional Attributes (R^2^ = 60.2%), indicating that sustainability and functionality account for a substantial portion of consumer perception. This explanatory power must nonetheless be read critically and in comparative perspective. An R^2^ of 29.4% exceeds the 10% minimum recommended for soft modeling [29] and approaches the moderate band of Chin’s classification [32], being also compatible with the medium-to-large range usual in consumer research [20]; values of this order are common in variance-based models of food and beverage consumer behavior, in which perceptions, emotions, habits, and context compete to explain evaluative outcomes, as illustrated by the MEC-based capsule-coffee model from which this study departs [14] and by behavioral applications of PLS-SEM in the coffee domain [9]. On one hand, the parsimonious chain Attributes → Benefits and Consequences → Perceived Value captures a meaningful, statistically robust share of value formation while omitting, by design, antecedents such as price perception, brand trust, consumption habit, and situational factors, whose inclusion is an avenue for model extension. On the other hand, the modest R^2^ of Convenient Benefits (18.5%) and of Benefits and Consequences (15.9%) indicates that convenience-related perceptions, in particular, are shaped by drivers beyond the cognitive chain modeled here, such as distribution channels, preparation equipment, and daily routines. Rather than overstating hypothesis confirmation, the results should be understood as evidence that the MEC is a necessary, but not exhaustive, mechanism of value formation in specialty coffee.

Moreover, all direct and indirect effects were statistically significant, confirming that Attributes influence Benefits and Consequences; that Benefits and Consequences influence Particular, Convenient, and Rational Benefits as well as Perceived Value; and that part of the impact of Attributes occurs through cognitive mediation.

This chain empirically confirms the logic Attributes → Consequences → Values proposed by Oliveira et al. [14], now extended through the explicit integration of sustainability, innovation, and quality dimensions.

The strongest indirect effect was observed for Rational Benefits (β = 0.323), suggesting that specialty coffee attributes, such as certified quality, origin, and sensory differentiation, activate perceptions of sustainability, socio-environmental responsibility, and economic rationality through consumers’ cognitive interpretation.

These findings empirically validate the central premise of the Means-End Chain theory: attributes do not automatically generate value; rather, they are cognitively interpreted and transformed into perceived consequences, which subsequently become internalized as values.

### 5.1. Managerial Implications

The results indicate that isolated attributes do not by themselves guarantee higher perceived value. What effectively generates value is the conversion of these attributes into benefits that are cognitively interpretable by consumers, consistent with the Attributes–Consequences–Values logic. In this sense, sensory quality must be communicated as an experience, sustainability must be translated into concrete, measurable impact, and innovation must be perceived as convenience and personalization. These findings converge with Giacalone et al. [8], Barahona et al. [10], and Carvalho et al. [11], who demonstrate that sensory and experiential aspects are interpreted multisensorially, encompassing not only chemical or technical characteristics but the entire consumption context. Thus, managers should structure the production chain as a chain of meaning rather than merely a supply chain, articulating technical attributes with narratives that activate perceived benefits.

The high R^2^ for Rational Benefits (65.6%), combined with the strongest indirect effect directed toward this dimension, indicates that sustainability is not a peripheral element but a structural driver of perceived value. As discussed by Van Keulen and Kirchherr [12] and Beske and Seuring [18], integrating sustainable practices throughout the value chain strengthens not only organizational legitimacy but also the product’s economic and ethical perceptions. Accordingly, mechanisms such as digital traceability, proactive transparency, communication of measurable environmental impact, and strategies aligned with circular economy principles are recommended. Given that the sample predominantly comprised individuals with higher levels of education and income, the findings are consistent with studies suggesting a greater propensity for sustainable consumption among consumers with higher educational capital, reinforcing the importance of strategically grounded sustainability communication.

Concerning innovation, Convenient Benefits showed moderate yet significant explanatory power, indicating that innovation operates not merely as a technological factor but as a cognitive facilitator of the consumption experience. Suryani et al. [7] demonstrate that innovation enhances organizational performance; in this study, it emerges as a link between attributes and personalized experience. Firms should therefore invest in differentiated brewing methods, sustainable capsule solutions, and technological integration within the consumption ritual, transforming innovation into a tangible experiential component.

Particular Benefits, explaining 37.5% of the variance, reveal that satisfaction, well-being, and emotional connection function as relevant mediators in value formation. Ramírez-Correa et al. [9] and Bartoloni et al. [13] indicate that enthusiasts and expert consumers seek authenticity and a symbolic connection with the product. This implies that brands should emphasize territorial storytelling, highlight origin and producer identity, and integrate consumers into the narrative of the production chain. In this context, specialty coffee transcends commodity status and becomes a cultural and identity-based practice. Translating these estimates into operational priorities for the specialty coffee industry, the results support the following sequence of actions. First, because the strongest path in the model links Benefits and Consequences to Rational Benefits (β = 0.810), investments with the highest expected return on perceived value are those that make sustainability verifiable: third-party certification, lot-level traceability with QR codes on packaging, and quantified environmental-impact communication. Second, the path to Particular Benefits (β = 0.612) justifies experience-centered actions, including guided cuppings, origin storytelling, and direct producer-consumer connections. Third, the path to Convenient Benefits (β = 0.430), combined with its modest R^2^, indicates that convenience alone does not differentiate, and should be deployed as a support strategy through subscription models, brewing guidance, and accessible preparation formats. Finally, because Benefits and Consequences transmit the entire effect of Attributes on Perceived Value (β = 0.542), communication should translate technical attributes, such as scores, varietals, and processing methods, into explicit benefit narratives rather than presenting them as raw specifications.

### 5.2. Theoretical Contributions

From a scientific perspective, the study empirically confirms the hierarchical structure of the Means-End Chain by demonstrating that attributes influence consequences, which in turn structure values, incorporating sustainability as a foundational rational dimension. The model proposed by Oliveira et al. [14] is thus extended by integrating innovation, sustainability, quality, and value chain dynamics within a unified structure validated through PLS-SEM. Furthermore, the study connects the sensory findings discussed by Giacalone et al. [8] and Carvalho et al. [11] with the sustainability and circular economy perspectives presented by Van Keulen and Kirchherr [12], thereby addressing an identified gap in the literature.

From an eco-humanistic perspective, the results indicate that specialty coffee consumption constitutes a relational practice connecting individuals, territory, production chains, sustainability, and social identity. Perceived value is not solely economic but symbolic and ethical. Coffee becomes a cultural artifact that translates socio-environmental responsibility into sensory experience.

In summary, value creation in specialty coffee is revealed to be multidimensional and systemic: attributes structure perceptions, benefits interpret attributes, and values emerge from symbolic conversion. Innovation reinforces sustainability, sustainability enhances quality, and quality consolidates value. Competitiveness in the specialty coffee sector ultimately depends on the capacity to transform technical excellence into human meaning, articulating operational performance and socio-environmental responsibility within an integrated value proposition.

## 6. Conclusions

This study explores how the different specialty coffee factors relate to consumer-perceived value. The findings show that product attributes do not generate value by themselves: their effect is fully transmitted through the benefits and consequences perceived by consumers, which unfold into rational, particular, and convenient benefits and consolidate into perceived value. The strongest structural effects were observed for Rational Benefits (H6, β = 0.810), followed by Particular Benefits (H5, β = 0.612) and Perceived Value (H7, β = 0.542). The objective of identifying, through a Means-End Chain model, the main linkages among attributes, benefits, and perceived value in specialty coffee was therefore achieved.

Theoretically, the study contributes quantitative evidence for the hierarchical logic of the Means-End Chain in the specialty coffee segment, extends the capsule-coffee model of Oliveira et al. [14] by incorporating sustainability, innovation, and quality dimensions, and characterizes the role of Benefits and Consequences as an indirect-only mediator. The prominence of the rational, sustainability-related dimension indicates that contemporary consumers of specialty coffee integrate ethical and environmental criteria into their purchasing decisions.

From a managerial perspective, and without repeating the detailed strategies presented in Section 5.1, the estimates indicate a sequence of priorities: making sustainability verifiable, investing in the consumption experience, and treating convenience as a supporting element rather than the main differentiator of specialty coffee.

The limitations of the study define the scope within which its conclusions apply. The sample was small (n = 88) and recruited through non-probabilistic convenience sampling with snowball dissemination, being concentrated in mature, highly educated, higher-income Brazilian consumers; the conclusions therefore apply to this niche of the Brazilian specialty coffee market and should not be extrapolated to the general population of coffee drinkers or to other countries. Consumption frequency was not used as a screening criterion, and the perspectives of producers, baristas, and industry professionals were not incorporated. All measures were self-reported perceptions collected online and are subject to self-selection bias; no objective sensory measurements, such as electronic tongue or electronic nose assessments, were employed, since the study focused deliberately on consumer perception rather than instrumental quality. Finally, the study did not address detailed technical aspects of cultivation nor the macroeconomic impacts of global trade policies on the specialty coffee market.

Future research should re-estimate the model with larger, preferably probabilistic samples, including frequent consumers, specialists, and industry professionals such as baristas and coffee graders, and should compare regions and countries to capture cultural and socioeconomic variation in value formation. Combining the perceptual model with objective sensory data, such as cupping scores and electronic tongue and electronic nose measurements, would make it possible to test how instrumental quality translates into perceived benefits. Finally, the inclusion of additional antecedents, such as price perception, brand trust, and consumption habit, and of moderators such as environmental awareness, may increase the explained variance of Perceived Value and Convenient Benefits reported here, while studies on digital traceability and production innovations may clarify emerging mechanisms of value creation along the chain.

## Figures and Tables

**Table 1:** Research instrument. Source: Authors’ own elaboration, adapted from Oliveira et al. [14].

Indicator	Description
**Intrinsic (Own) Attributes (AP)**	
AP1	The flavor of the final product of specialty coffee is superior.
AP2	Consuming a product with a high quality rating is important to me.
**Functional Attributes (FA)**	
AF1	The market offers a wide variety of specialty coffees.
AF2	I have easy access to specialty coffees.
AF3	Specialty coffee is easy to handle.
**Convenient Benefits (IN)**	
IN1	I perceive transparency in the production processes of specialty coffee.
IN2	Specialty coffees encourage me to experiment with different brewing methods.
IN3	I feel like I’m having a personalized experience when drinking specialty coffees.
**Particular Benefits (QU)**	
QU1	It’s easy to prepare specialty coffees.
QU2	Specialty coffees allow me to drink different varieties of coffee.
QU3	Specialty coffees have different flavor profiles.
QU4	Specialty coffee brings me closer to the coffee producer.
QU5	Specialty coffees allow me to drink coffees from different origins.
QU6	Specialty coffee engages me socially.
**Rational Benefits (SU)**	
SU1	By consuming specialty coffees, I am contributing to the environment.
SU2	When I drink specialty coffee, I feel a greater moral awareness.
SU3	Specialty coffee allows me to avoid waste.
SU4	By consuming specialty coffees, I promote cooperation and collaboration.
**Perceived Value (V)**	
V1	Specialty coffee allows me to live my life well.
V2	Drinking specialty coffee makes me feel more confident.
V3	I feel fulfilled when I drink specialty coffee.
V4	When I drink specialty coffee, I have nostalgic moments.
V5	I can be helpful when consuming specialty coffee.
V6	Drinking specialty coffee brings me satisfaction.
V7	My friends recognize me socially because I drink specialty coffee.
V8	My relatives and friends give me more confidence and credibility because I drink specialty coffees.

Note: Bold text indicates the latent variable or construct under which the corresponding measurement items are grouped.

**Table 2:** Internal consistency, reliability, and AVE indicators. Source: Authors’ own elaboration, extracted from SmartPLS 4.1.1.8 (2026).

	rho_c	AVE
1. Attributes	0.736	0.582
2. Functional attributes	0.842	0.640
3. Own attributes	0.829	0.707
4. Convenient benefits	0.875	0.778
5. Benefits and consequences	0.827	0.705
6. Specific benefits	0.838	0.633
7. Rational benefits	0.903	0.701
8. Perception of Value	0.888	0.533

**Table 3:** HTMT results. Source: Authors’ own elaboration, extracted from SmartPLS 4.1.1.8 (2026).

Heterotrait-Monotrait Ratio (HTMT)	
Own Attributes <-> Functional Attributes	0.264
Convenient Benefits <-> Attributes	0.875
Convenient Benefits <-> Functional Attributes	0.137
Convenient Benefits <-> Own Attributes	0.811
Benefits and Consequences <-> Functional Attributes	0.491
Benefits and Consequences <-> Own Attributes	0.429
Benefits and Consequences <-> Convenient Benefits	0.630
Particular Benefits <-> Attributes	0.784
Particular Benefits <-> Functional Attributes	0.379
Particular Benefits <-> Own Attributes	0.392
Particular Benefits <-> Convenient Benefits	0.606
Rational Benefits <-> Attributes	0.503
Rational Benefits <-> Functional Attributes	0.266
Rational Benefits <-> Own Attributes	0.195
Rational Benefits <-> Convenient Benefits	0.204
Rational Benefits <-> Particular Benefits	0.194
Perception of Value <-> Attributes	0.843
Perception of Value <-> Functional Attributes	0.173
Perception of Value <-> Own Attributes	0.740
Perception of Value <-> Convenient Benefits	0.705
Perception of Value <-> Particular Benefits	0.349
Perception of Value <-> Rational Benefits	0.411

**Table 4:** Hypothesis testing. Source: Authors’ own elaboration, extracted from SmartPLS (2026).

Hypotheses	Beta	%	T-Value	Confidence Interval		Significant
				2.5%	97.5%	
Attributes -> Functional Attributes	0.776 ***	60.22%	12.689	0.672	0.848	Yes
Attributes -> Own Attributes	0.750 ***	56.25%	9.870	0.608	0.832	Yes
Attributes -> Benefits and Consequences	0.399 ***	15.92%	4.563	0.225	0.565	Yes
Benefits and Consequences -> Convenient Benefits	0.430 ***	18.49%	4.942	0.269	0.608	Yes
Benefits and Consequences -> Particular Benefits	0.612 ***	37.45%	10.518	0.492	0.715	Yes
Benefits and Consequences -> Rational Benefits	0.810 ***	65.61%	14.183	0.676	0.884	Yes
Benefits and Consequences -> Perceived Value	0.542 ***	29.38%	8.217	0.425	0.681	Yes

* *p* < 0.05; ** *p* < 0.01; *** *p* < 0.001/VIF ≤ 3.3.

**Table 5:** Indirect effects. Source: Authors’ own elaboration, extracted from SmartPLS (2026).

Indirect Effect	Beta	T-Value	*p*-Value
Attributes -> Benefits and consequences -> Particular benefits	0.244	3.841	0.000
Attributes -> Benefits and consequences -> Rational benefits	0.323	4.123	0.000
Attributes -> Benefits and consequences -> Perception of value	0.216	3.759	0.000
Attributes -> Benefits and consequences -> Convenient benefits	0.171	3.323	0.001

## Data Availability

The data presented in this study are available on request from the corresponding author. The data are not publicly available due to privacy and ethical restrictions, as they were collected from human participants and may contain information that could allow indirect identification when combined with demographic or contextual variables.

## Appendix A

Table A1 reports the outer loadings of the reflective measurement model in the initial estimation (Model 1, 26 items) and in the final, purified model (Model 2, 21 items), complementing the aggregate reliability and validity statistics presented in Section 4.1. Indicators IN1, QU1, QU4, and QU6 were removed owing to insufficient indicator reliability, and indicator V8 was excluded during instrument refinement.

**Table A1:** Outer loadings in the initial and final measurement models. Source: Authors’ own elaboration, extracted from SmartPLS (2026).

Construct	Item	Outer Loading (Initial Model)	Outer Loading (Final Model)
Intrinsic (Own) Attributes (AP)	AP1	0.842	0.844
	AP2	0.840	0.838
Functional Attributes (AF)	AF1	0.812	0.814
	AF2	0.830	0.832
	AF3	0.756	0.752
Convenient Benefits (IN)	IN1	0.436	Removed
	IN2	0.823	0.911
	IN3	0.812	0.852
Particular Benefits (QU)	QU1	0.256	Removed
	QU2	0.676	0.801
	QU3	0.593	0.819
	QU4	0.578	Removed
	QU5	0.625	0.766
	QU6	0.699	Removed
Rational Benefits (SU)	SU1	0.859	0.862
	SU2	0.865	0.866
	SU3	0.774	0.762
	SU4	0.847	0.854
Perceived Value (V)	V1	0.775	0.778
	V2	0.829	0.831
	V3	0.829	0.815
	V4	0.684	0.688
	V5	0.673	0.693
	V6	0.673	0.644
	V7	0.633	0.637
	V8	—	Excluded
